# Geometric and Colour Data Fusion for Outdoor 3D Models

**DOI:** 10.3390/s120606893

**Published:** 2012-05-25

**Authors:** Pilar Merchán, Antonio Adán, Santiago Salamanca, Vicente Domínguez, Ricardo Chacón

**Affiliations:** 1 Industrial Engineering School, University of Extremadura, Avda. de Elvas, s/n, 06071 Badajoz, Spain; E-Mail: ssalamanca@unex.es; 2 School of Computer Engineering, University of Castilla La Mancha, Ronda de Calatrava, 5,13071 Ciudad Real, Spain; E-Mails: Antonio.Adan@uclm.es (A.A.); Vicente.Dominguez@uclm.es (V.D.); Ricardo.Chacon@uclm.es (R.C.)

**Keywords:** 3D modelling, texture fusion, 3D digitalization, range data

## Abstract

This paper deals with the generation of accurate, dense and coloured 3D models of outdoor scenarios from scanners. This is a challenging research field in which several problems still remain unsolved. In particular, the process of 3D model creation in outdoor scenes may be inefficient if the scene is digitalized under unsuitable technical (specific scanner on-board camera) and environmental (rain, dampness, changing illumination) conditions. We address our research towards the integration of images and range data to produce photorealistic models. Our proposal is based on decoupling the colour integration and geometry reconstruction stages, making them independent and controlled processes. This issue is approached from two different viewpoints. On the one hand, given a complete model (geometry plus texture), we propose a method to modify the original texture provided by the scanner on-board camera with the colour information extracted from external images taken at given moments and under specific environmental conditions. On the other hand, we propose an algorithm to directly assign external images onto the complete geometric model, thus avoiding tedious on-line calibration processes. We present the work conducted on two large Roman archaeological sites dating from the first century A.D., namely, the Theatre of Segobriga and the *Fori Porticus* of Emerita Augusta, both in Spain. The results obtained demonstrate that our approach could be useful in the digitalization and 3D modelling fields.

## 1. Introduction

The construction of geometrical outdoor models, which consist of millions of points and patches, can be carried out with relatively efficiency and accuracy by using current 3D sensors, particularly with phase-shift and time of flight scanners. Colour information can also be acquired with high resolution by using appropriate digital cameras and lenses, which lead to a wide variety of parameters that can be set by hand.

However, the combination of a set of 3D clouds of points and colour images to obtain a photorealistic model is still an area of research which has a number of unsolved problems. The first of these relates to the experimental setup itself. Some scanners have an on-board camera with determined technical characteristics, and others permit the attachment of an external camera that has to be fitted in a specific position. Thus, in the field of digitalization, geometrical information and colour information are usually captured at the same time, under the illumination conditions that exist at the very moment of the 3D data acquisition. This makes the fusion of both types of information highly prone to errors. Under these circumstances, the process of geometrical and colour data acquisition is subdued in terms of both space and time: in terms of space because the sensor and the camera take up established positions during the data acquisition stage, and in terms of time because the geometrical information and the colour information are sensed simultaneously.

Consequently, mismatches commonly occur when combining texture images with 3D geometrical data either due to parallax effects that depend on the baseline between the laser scanner and the optical centre of the camera, or to differences between several colour images taken at different times and, therefore, under different illumination conditions.

The second problem arises when attempting to register and merge the set of data acquired from different positions in a unique representation model. Geometrical registration and 3D data integration problems, of course, were solved a number of years ago. There are numerous approaches in the literature related to registration, many of them based on the original well-known algorithm by Besl and McKay known as ICP (Iterative Closest Point Algorithm) [1]. Zhang presents [2] an important variant of the original algorithm. Xiao *et al.* [3] modify the ICP and utilize both regional surface properties and shape rigidity constraint to align a partial object surface and its corresponding complete surface. In addition to ICP, some other methods have been proposed for 3D registration. For example, in [4] genetic algorithms are used for registration of 3D data with low overlap and which may include substantial noise. Extensive comparison of registration methods can be seen in [5,6].

For the resolution of the integration problem, several methods have also been proposed. The most important classic ones are those based on “zippering” [7] or volumetric [8] algorithms. In [9] a faster variant of zippering algorithm is presented. Santos *et al.* propose in [10] a high fidelity merging algorithm that it is used for digital art preservation. Nevertheless, the integration of colour information taken from different viewpoints is an issue that still requires further research.

The problem in texture fusion is mainly caused by the colour disparity between two images. This is brought about by several reasons: First, changes in the angle between the observer direction and the normal of the reflecting surface and, second, variations in illumination conditions in the environments where they were captured. The third reason concerns the camera parameters, which may change from one scan to the next.

Many researchers have proposed solutions based on pairwise colour correction, colour mixing algorithms and image illumination change detection. When the scene is an artificially illuminated interior the difficulty for texture fusion is lessened, and acceptable results are obtained in most of the cases. In some cases, the light of the scene is controlled, so it is assumed that the scene is scanned under constant illumination. This assumption greatly simplifies the colour integration method which consists merely of searching for optimal blending functions [11–14]. For non-controlled scenarios, researchers have come up with only partial solutions. Most of these solutions are based either on global approaches, in which the complete colour image from one position of the scanner is processed (and therefore, modified), or on local strategies, in which only the colours at certain points of the image are corrected. In [15] the colours are inserted by detecting featured points in the image and defining the overlapped regions. Park *et al.* [16] developed a method that simultaneously fills in holes and associates (not acquired) colour. The study in [17] deals with several images of the scene, which are taken under different light conditions, and corrects discontinuities in the overlapped zones. Troccoli *et al.* [18] compute a relighting operator by analysing the overlapped area of a pair of images: the source image to be relighted and a target image. They then apply this operator over the non-overlapping region of the source, making it consistent with the target image. In [19], Dellepiane *et al.* present a colour correction space which is capable of providing a space-dependent colour correction for each pixel of the image. It estimates the flash light position and permits identification and removal of annoying artifacts, such as highlights and shadows. Since many of the developed methodologies for the mapping of colour information on 3D models also include the possibility of discarding parts of the input images or of selectively assigning a weight to contributing pixels, these authors state that their proposal is an interesting addition to such methods

Drastic approaches can be also found. For instance Sun *et al.* [20] remove, in overlapped regions, the less confident triangles and consider only the texture of the selected patches, avoiding any colour fusion procedure. Bernardini *et al.* [21] adjust the colours by global colour registration, imposing a restriction that distributes error in the results equally on the model. Finally, Xu *et al.* [22] integrate texture maps of large structures by decomposing the colour into two frequency bands and combining them.

The other line of research focuses on the obtention of a surface reflectance map of the scene. In this, the 3D model is illumination-free and can be rendered under any illumination conditions. Some interesting methods of obtaining a reflectance function are presented in [23] and [24]. These proposals were developed under laboratory conditions in which light positions are assumed to be known. Later, Debevec *et al.* [25] estimated the spatially-varying surface reflectance of complex scenes under natural illumination conditions. This approach uses a novel lighting measurement apparatus to record the full dynamic range of both sunlit and cloudy natural illumination conditions. Finally, in [26] the authors present a model of natural illumination as a combination of point source plus ambient component. They are able to compute the actual reflectance and to remove any effects of illumination in the images.

In spite of researchers' efforts over the last decade, the generated models are frequently incomplete or have neither sufficient accuracy nor quality. In particular, when dealing with outdoor scenarios the process becomes much harder. The colour images are severely affected by weather variability (rain and humidity) as well as the time of day at which they are taken. Furthermore, differing orientation and placement of the camera with respect to the surface must once again be taken into account.

This paper addresses the problem of how to build complete photorealistic models by uncoupling the time and particular circumstances in which 3D data and colour of the scene are captured. This issue is tackled from two different perspectives. Firstly, using a complete model (geometry plus texture) obtained after several data acquisition sessions, our aim is to modify the original texture provided on site by the scanner on-board camera with the colour information extracted from external images taken at particular times of day, times of the year (season) or under more suitable environmental conditions. We discuss this in Section 2. Secondly, we aim to keep the processes of geometrical data acquisition and colour acquisition apart, so that they become independent. Thus, the acquisition of colour information with external high-resolution cameras could be executed at chosen times, separately from the data acquisition for the geometrical modelling process, which is not sensitive to time or weather variations. This task therefore involves assigning colour to the geometrical model once it is finished, rather than modifying an existing colour model on it. Section 3 of this paper explains the procedure carried out to do this.

A further aspect to bear in mind with regard to our study relates to the fact that our algorithms increase the automation level in the overall geometry and colour merging process. In particular, we aim to automate two sub-processes: the colour fusion algorithm for several views by using a previous analysis of the global colour of the scene, and the search for the best view in the stage of making the match between the model and the external images. The automation of these phases is intended to go beyond manual and user dependent procedures. Despite this, the current state of the method presented in this paper requires user intervention in some steps. For instance, the user selects the set of external images to be used and can also vary certain specific parameters at the colour integration stage, such as window sizes, masks, thresholds, *etc.* These aspects are dealt with in Section 4.

Finally, the experimental section shows the results obtained in large outdoor scenarios. Specifically, we present the work carried out in two Roman archaeological sites that date back to the first century A.D., the theatre of Segobriga and the *Fori Porticus* of Emerita Augusta, both in Spain.

## 2. Colour Modification on Synthetic Models

Let us assume a 3D model ***M*** of an object obtained from the information acquired with a scanner equipped with an internal low-resolution camera. The rough information provided by the sensor is composed of the 3D coordinates of the points of the object and their corresponding colors in RGB format. A meshing algorithm then yields a patch representation, including vertices and normal vectors. We specifically used the volumetric approach published in [8] to generate the partial meshes. The view registration and geometric integration problem is solved using the k–d tree algorithm published by Zhang in [2]. Given the model ***M*** (***V, F, N, C***) where:

***V* =** {***V*_1_, *V*_2_**,**…, *V*_n_**} are the 3D points of the model, that is, the vertices of the final mesh.
***F* =** {***F*_1_, *F*_2_**,**…, *F*_n_**} are the patches of the mesh model.
***N* =** {***N*_1_, *N*_2_**,**…, *N*_n_**} are the normal vectors for each vertex of the mesh model.
***C* =** {***C*_1_, *C*_2_**,**…, *C*_n_**} are the colour components for each vertex of the mesh model.

**Figure 1.**
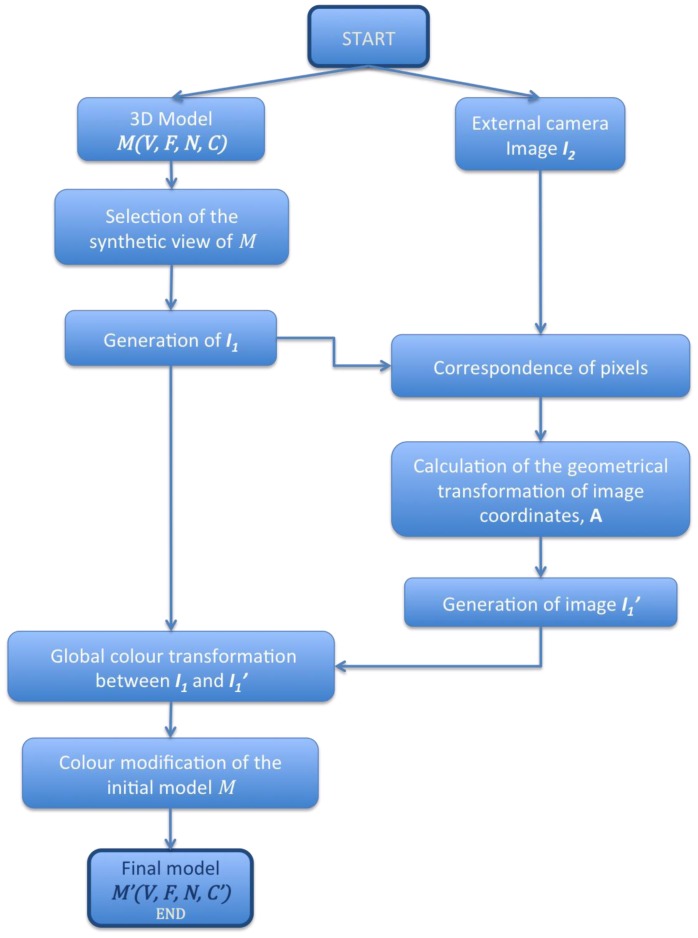
Sketch of the procedure used to modify the colour of a 3D model.

Our goal is to modify ***C*** by using a new set of images taken with external high-quality cameras and to obtain a photo-realistic model ***M*′** (***V, F, N, C***).We achieve this after the sequence of steps drawn in the sketch shown in Figure 1.

**Figure 2.**
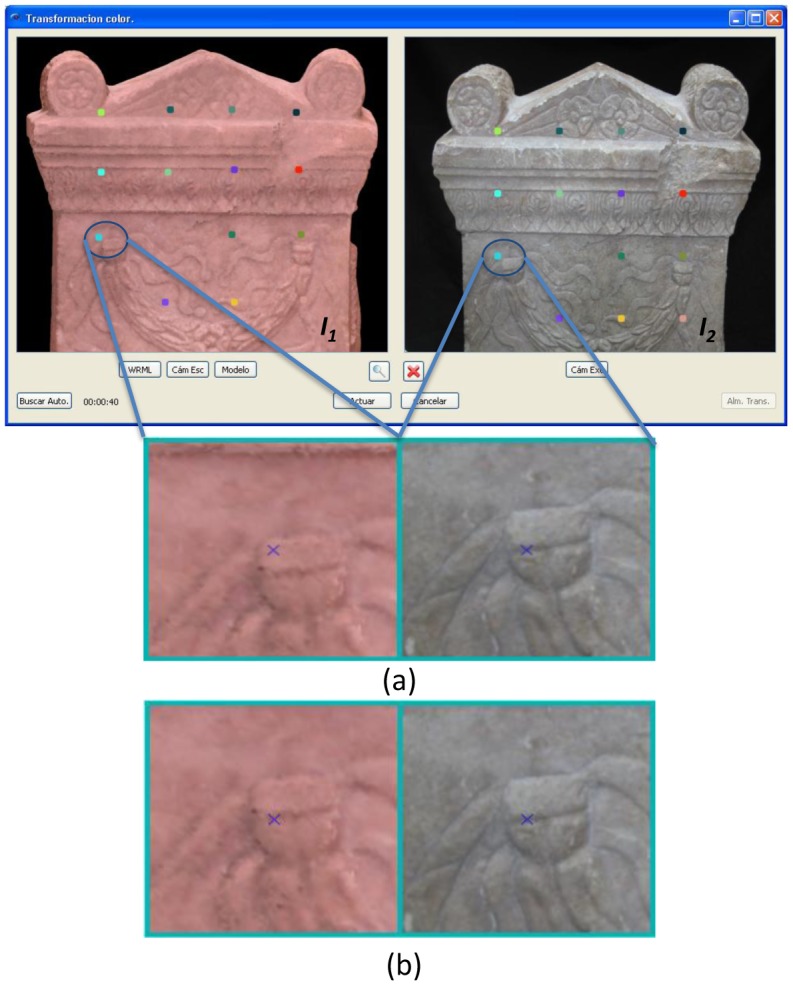
(**a**) Selection of relevant pixels in images ***I****_1_* (taken from the coloured model) and ***I****_2_* (external image) and one pair of pixels chosen to run the algorithm of error minimization; (**b**) Corrected position of the chosen pixel in ***I****_1_* depicted *versus* the position of its related pixel in ***I****_2_*.

In the first step, we choose two images, ***I****_1_* and ***I****_2_*. ***I****_1_* can be either the image provided by the camera of the scanner itself or an ortho-photo of ***M*** from a viewpoint close to the viewpoint of the external high-quality camera when taking ***I****_2_*. The following step consists of selecting control points in both images. In the software we have created to execute colour modification, these can be marked automatically or by hand. If the automatic option is selected, the algorithm picks several pixels equally separated in the image ***I****_2_* and looks for their corresponding ones in ***I****_1_*. Then, for every pair of marked pixels, an iterative algorithm is run so that the position of each selected pixel in ***I****_1_* is recalculated, and the correspondence error between pixels is minimized. The procedure for the iterative algorithm can be summarized as follows: for a given pair of pixels, a window *W_2_* centred in the corresponding pixel from ***I****_2_* is set. At the same time, a smaller window *W_1_* is selected for the pixel in ***I****_1_*. By means of a cross-correlation algorithm all the possible superimpositions of *W_1_* onto *W_2_* are obtained. The cross-correlation factor *R* is calculated by following Equation (1).

(1) $$R=\frac{\sum_{x}\sum_{y}(P_{1,xy}-{\bar{I}}_{1})(P_{2,xy}-{\bar{I}}_{2})}{\sqrt{\left(\sum_{x}\sum_{y}{\left(P_{1,xy}-{\bar{I}}_{1}\right)}^{2})(\sum_{x}\sum_{y}{\left(P_{2,xy}-{\bar{I}}_{2}\right)}^{2}\right)}}$$

where P_1_,*_xy_* is the pixel (*x*,*y*) in the image ***I****_1_*, P_2_,*_xy_* is the pixel (*x*,*y*) in ***I****_2_* and ***Ī*_1_** and ***Ī*_2_** are the arithmetic means of the components of ***I****_1_* and ***I****_2_*, respectively. As is known, the superimposition that gives the cross-correlation factor closest to 1 corrects the correspondence of pixels between ***I****_1_* and ***I****_2_*. Figure 2 shows an example detailing several stages of the pixels correspondence process.

The next step consists of correcting the original image ***I****_1_*. The correspondence between *n* pixels from ***I****_1_* and ***I****_2_* can be expressed by means of a homogeneous transformation **A** in Equation (2):

(2) $$P_{e}=AP_{m}$$

where ***P****_e_* and ***P****_m_* are the coordinates of the pixels in ***I****_1_* and ***I****_2_*, respectively.

Note that the alignment between images ***I****_1_* and ***I****_2_* is not an affine homography because the scene is not exactly the same for both cases. ***I****_1_* and ***I****_2_* are taken for different scenes and seen under different optical properties. Thus, ***I****_1_* corresponds to the image of a 3D model and is generated under graphical characteristics. In this case, the scene is a 3D model. Nevertheless, ***I****_2_* is the real image captured by the camera, which has its own optical properties, and the scene is now the real environment.

For a general case:

(3) $$P_{e}=\left(\begin{array}{cccc} \lambda_{1}x_{e1} & \lambda_{1}x_{e2} & \cdots & \lambda_{n}x_{en}\\ \lambda_{1}y_{e1} & \lambda_{1}y_{e2} & \cdots & \lambda_{1}y_{en}\\ \lambda_{1} & \lambda_{2} & \cdots & \lambda_{n} \end{array}\right),A=\left(\begin{array}{ccc} a_{11} & a_{12} & a_{13}\\ a_{21} & a_{22} & a_{23}\\ a_{31} & a_{32} & a_{33} \end{array}\right),P_{m}=\left(\begin{array}{cccc} x_{m1} & x_{m2} & \cdots & x_{mn}\\ y_{m1} & y_{m2} & \cdots & y_{mn}\\ 1 & 1 & \cdots & 1 \end{array}\right)$$

If we chose a_33_ = 1, then:λ_1_=*a*_31_*x*_*m*1_+*a*_32_*y*_*m*1_+1λ_2_=*a*_31_*x*_*m*2_+*a*_32_*y*_*m*2_+1⋮λ_*n*_=*a*_31_*x_mn_*+*a*_32_*y_mn_*+1

Taking these *n* related points as a starting point, Equation (2) leads to the expression:

(4) $$HA_{v}=X_{p}$$

and matrices ***X****_p_* and ***H*** are obtained from **P***_e_* and **P***_m_* in the following way:

(5) $$X_{p}=\left(\begin{array}{l} x_{e1}\\ y_{e1}\\ x_{e2}\\ y_{e2}\\ x_{e3}\\ y_{e3}\\ \cdots \cdots \\ x_{en}\\ y_{en} \end{array}\right),H=\left(\begin{array}{ccccccc} x_{m1} & y_{m1} & 1 & \begin{array}{cc} 0 & 0 \end{array} & 0 & -x_{m1}x_{e1} & -y_{m1}x_{e1}\\ \begin{array}{cc} 0 & 0 \end{array} & 0 & x_{m1} & y_{m1} & 1 & -x_{m1}y_{e1} & -y_{m1}y_{e1}\\ x_{m2} & y_{m2} & 1 & \begin{array}{cc} 0 & 0 \end{array} & 0 & -x_{m2}x_{e2} & -y_{m2}x_{e2}\\ \begin{array}{cc} 0 & 0 \end{array} & 0 & x_{m2} & y_{m2} & 1 & -x_{m2}y_{e2} & -y_{m2}y_{e2}\\ \ldots \ldots & \ldots \ldots & \ldots \ldots & \ldots \ldots & & \begin{array}{cc} \ldots \ldots & \ldots \ldots \end{array} & \begin{array}{cc} \ldots \ldots & \ldots \ldots \end{array}\\ x_{mn} & y_{mn} & 1 & \begin{array}{cc} 0 & 0 \end{array} & 0 & -x_{mn}x_{en} & -y_{mn}x_{en}\\ \begin{array}{cc} 0 & 0 \end{array} & 0 & x_{mn} & y_{mn} & 1 & -x_{mn}y_{en} & -y_{mn}y_{en} \end{array}\right),A_{v}=\left(\begin{array}{c} a_{11}\\ a_{12}\\ a_{13}\\ a_{21}\\ a_{22}\\ a_{23}\\ a_{31}\\ a_{32} \end{array}\right)$$

Then we solve the Equation (4) and obtain the transformation matrix between the two images ***I****_1_* and ***I****_2_*:

(6) $$A_{v}={\left(H^{T}H\right)}^{-1}H^{T}X_{p}$$

Once we have **A**, the final corrected image, $I_{1}^{\prime }$ will be the one obtained after modifying the RGB components with the RGB values of the corresponding pixels in the image ***I****_2_*. Let us denote ***C*** as the vector which contains the three colour components, ***C*** = [*R G B*]*^t^*. For each pixel of ***I****_1_* the colour is modify as follows:

(7) $$\begin{array}{l} C(P_{k}^{\prime })=C(P_{k})\\ P_{k}=\frac{AP_{k}^{\prime }}{\lambda_{k}},\forall k \end{array}$$

*P_k_* and $P_{k}^{\prime }$ being the coordinates of the *k*-th pixel in ***I****_2_* and $I_{1}^{\prime }$, respectively, and *C*(*P_k_*) being the colour information stored in the *k*-th pixel of ***I****_2_* and $C(P_{k}^{\prime })$ the colour information assigned to the *k*-th pixel in $I_{1}^{\prime }$.

In those cases when the image deformations are not considered, that is, when dealing with a geometric transformation, the following equalities are verified:

(8) $$\begin{array}{c} \lambda_{1}=\lambda_{2}=\cdots =\lambda_{k}=\cdots =\lambda_{n}=1;\\ a_{31}=a_{32}=0;a_{33}=1 \end{array}$$

At the end of this process, a global correction algorithm is applied to the initial image ***I****_1_*. Thus, the transformation that relates the colour components of image ***I****_1_* to the colour components of image $I_{1}^{\prime }$ can be modelled by a linear transformation as follows:

(9) $$C_{I_{1}^{\prime }}=ZC_{I_{1}}$$

and the values of the elements of **Z** can be computed using Equation (10):

(10) $$Z=\left({\text{C}}_{I_{1}^{\prime }}{C_{I_{1}}}^{T}\right){\left(C_{I_{1}^{\prime }}{C_{I_{1}}}^{T}\right)}^{-1}$$

Note that this section is developed taking one view of the original coloured model and therefore **Z** represents a colour transformation for this particular view. Nevertheless, in the case of uniform colour models, transformation **Z** could be used to modify the colour of any partial view of the model. This circumstance frequently occurs in heritage pieces, like marble statues or stone monuments. Formally:

(11) $$C_{M\prime }=ZC_{M}$$

**Figure 3.**
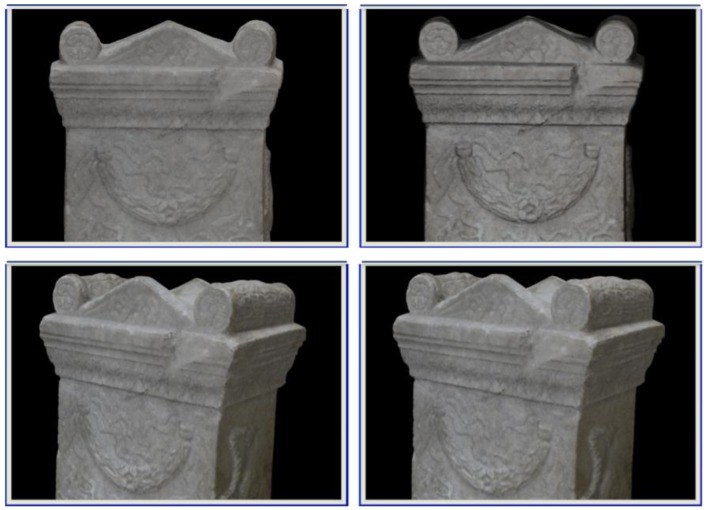
Correction of texture: (**left**) Corrected model assuming a pure geometric transformation between *I_1_* and *I_2_*; (**right**) Result by considering image deformations.

Figure 3 shows an example in which the colour of the whole model of the piece has been modified.

## 3. Applying External Images onto the Geometrical Models

Let us now suppose that we have a geometrical model ***M*** (***V, F, N***) of a large scenario with no integrated texture and a set of high resolution images taken without any restrictions with an external camera. Restrictions concern any aspect related to the image (size, accuracy, resolution…) and the time at which the images were taken. With the collection of images and the geometry of the scene, a procedure to obtain a final complete model that comprises colour and geometry has been developed.

It is well known that colour assignation can be carried out on-line using a camera and always under an accuracy camera-scanner calibration. As mentioned in Section 1, this involves certain inconveniences in colour integration post-processing. By decoupling geometry and colour with respect to the moment at which the data are taken, our aim is to integrate the texture in the geometry in a controlled off-line process.

Our procedure consists of three main stages:

1. Images matching. We look for a viewpoint in the geometrical model to generate an ortho-image as similar as possible to an external image.
2. Generation of the coloured ortho-image.
3. Colour assignation into the geometrical model.

## 3.1. Images Matching

**Figure 4.**
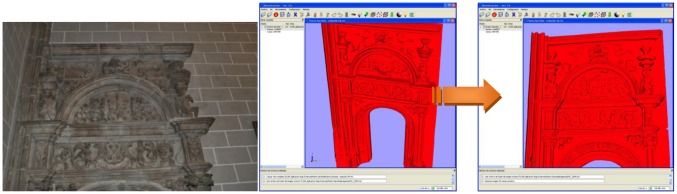
(**left**) External image ***I****_2_*; (**right**) Uncoloured 3D model and chosen view to obtain image ***I****_1_*.

At this first stage, two images are chosen: an ortho-photo of the 3D geometrical model, which will be denoted as ***I****_1_*, and an external image acquired without any restrictions, ***I****_2_*. The viewpoint that provides the image ***I****_1_* must be defined in such a way that the resulting projected view can be compared to the external image ***I****_2_*, as shown in Figure 4. Several transformation matrixes must be stored when projecting the model to assure the reverse transformation from the coloured ortho-image to the 3D model, *i.e.*, the matrix that controls the position of the viewpoint which the user views the model from, the projection transformation matrix, which defines the kind of perspective applied to the 3D model (either orthogonal or orthographical), and the model rotation/translation matrix that stores the rotation and/or translation undergone by the 3D model to be placed in a view close to the external image view. It is also necessary to record the size (in pixels) of the window in which the 3D model is viewed.

### 3.2. Generation of the Coloured Ortho-Image

To begin this stage, we establish a correspondence between images **I_1_** and **I_2_** by marking corresponding pixels manually. These sets of points define two matrixes of 2D coordinates, denoted as P_m_ and P_e_, can be written as:

$$P_{m}=\left(\begin{array}{c} \begin{array}{cc} {\text{x}}_{m1} & y_{m2} \end{array}\\ \begin{array}{cc} {\text{x}}_{m2} & {\text{y}}_{m2} \end{array}\\ \begin{array}{cc} x_{m3} & {\text{y}}_{m3} \end{array}\\ \ldots \ldots \\ \begin{array}{cc} x_{mn} & y_{mn} \end{array} \end{array}\right);P_{\text{e}}=\left(\begin{array}{c} \begin{array}{cc} x_{\text{e}1} & y_{\text{e}2} \end{array}\\ \begin{array}{cc} x_{e2} & y_{e2} \end{array}\\ \begin{array}{cc} x_{e3} & y_{e3} \end{array}\\ \ldots \ldots \\ \begin{array}{cc} x_{\text{en}} & y_{en} \end{array} \end{array}\right)$$

The transformation between the two images is modelled as:

(12) $$P_{e}=AP_{m}$$

with **A** being a transformation matrix *3x3* that includes the rotation, translation and deformation of image ***I****_1_*. We find the values of **A** by carrying through a sequence of operations as described in Section 2 (Equations (3–6)). We then build a new image with the RGB components of external image ***I****_2_* and matrix **A**, that is, we build a new image $I_{1}^{\prime }$, in which the pixels have the colour of their corresponding pixels in ***I****_2_*, as expressed in Equation (7).

**Figure 5.**
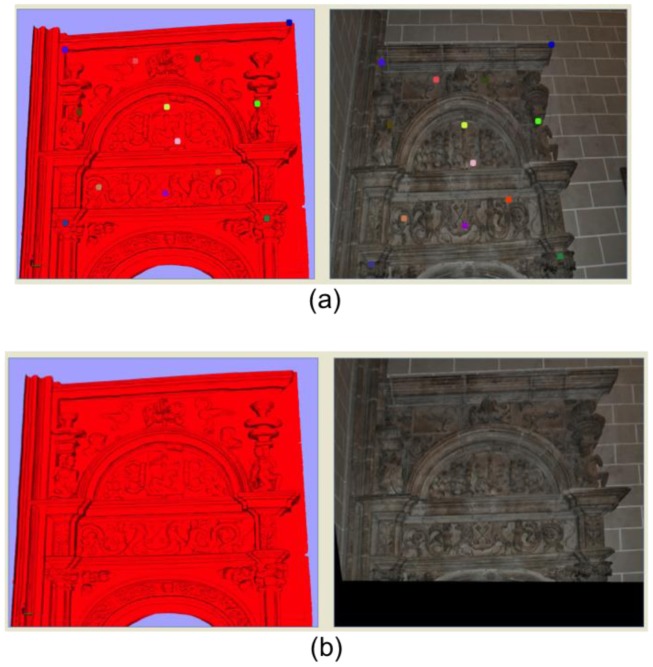

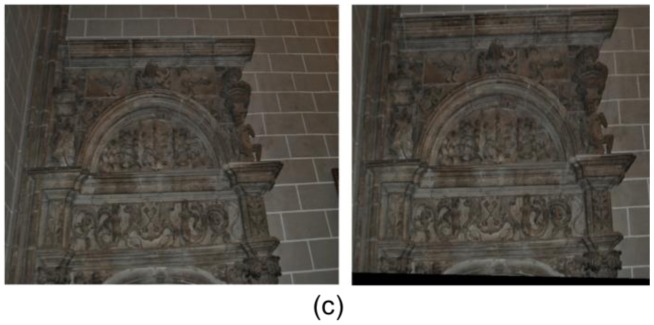
Generation of the coloured ortho-image: (**a**) Selection of the corresponding pairs of points onto the 3D geometrical model ortho-image and the external image; (**b**) Result of the colour transformation method on ***I****_1_* (left) to obtain $I_{1}^{\prime }$ (right); (**c**) Comparison between the original external image ***I****_2_* (left) and the generated coloured ortho-image $I_{1}^{\prime }$ (right).

Figure 5 displays some stages of this procedure, such as the selection of the corresponding points in both the projected image and the external one, or the generated coloured ortho-image achieved in the end.

### 3.3. Colour Assignation in the Geometric Model

Once we have the coloured image generated in the preceding step, we look for the correspondence between each pixel in image $I_{1}^{\prime }$ and the points of the 3D geometrical model. To do so, we start by restoring the point of view of ***M***. The basic idea is to recover the size and placement in which the ortho-image ***I****_1_* was taken. Therefore, we need the information in the different matrixes stored previously.

**Figure 6.**
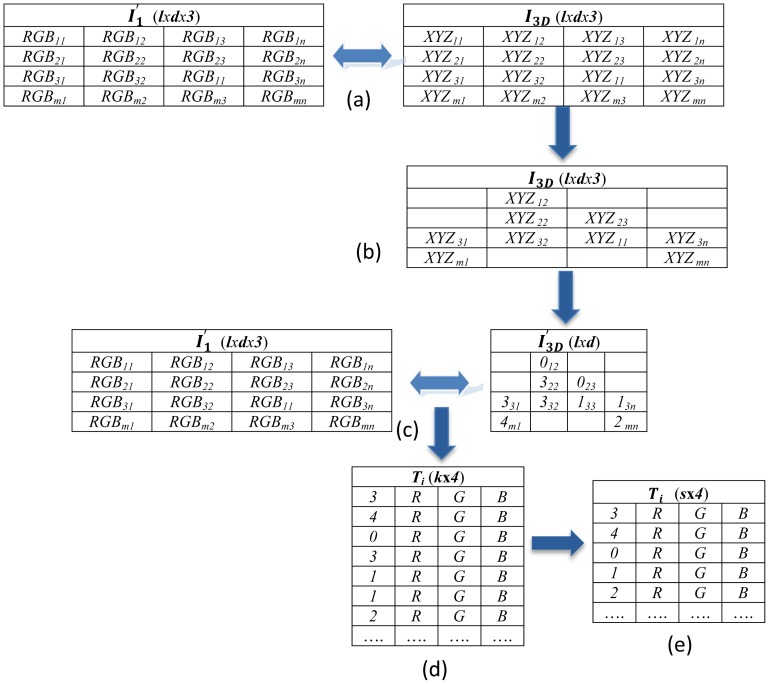
(**a**) Mapping of the 2D information in the 3D coordinates; (**b**) Elimination of the point coordinates that do not belong to the geometrical model; (**c**) Definition of the vertex indexes associated to the model; (**d**) Storage of the vertex indexes and their associated colour; (**e**) Assignation of a unique colour to each vertex in the geometrical model.

As is known, the description of a 3D object can be given by a list of 3D coordinates (points), and the colour associated to these points. An optional point indexation to form faces can also exist. In order to map the image $I_{1}^{\prime }$ onto the model ***M***, we use a function which maps the 2D coordinates of a graphical window to the corresponding 3D coordinates of the model. It generates a matrix ***I*_3_***_D_* in which every pixel in the image has an associated point with 3D coordinates. Figure 6(a) illustrates this step. It is understood that the value of the 3D coordinates is calculated by interpolation on a mesh model, but the assignation must be necessarily made to a vertex of the geometrical mesh, as will be discussed below. The matrix ***I*_3_***_D_* is subjected to a cleaning process to eliminate those elements which have no associated 3D components, in order to purge the assignation of pixels that corresponds to the background of the image $I_{1}^{\prime }$ (Figure 6(b)). For every valid element in matrix ***I*_3_***_D_*, the closest vertex is sought in the mesh model, generating a new matrix $I_{3D}^{\prime }$ that consists of the vertex indexes associated to the model (Figure 6(c)). The matrixes $I_{1}^{\prime }$ (***l****x****d****x****3***) and $I_{3D}^{\prime }$ (***l****x****d***) are then combined in a unique matrix ***T****_i_* (***k****x****4***) which contains the vertex indexes of the 3D model and their associated colour in the image $I_{1}^{\prime }$ (Figure 6(d)). Since it may be the case that the same node has more than one colour assigned, the matrix ***T****_i_* is resized so that each vertex in the model has a unique associated colour, this colour being the mean of the possible colour values assigned in matrix ***T****_i_* (Figure 6(e)).

## 4. Multi-View Colour-Fusion Algorithm

When the colour assignation for a specific viewpoint is finished, we have a coloured model ***M*** (***V, F, N, C*′**) in which only a part of its vertices has colour information. In order to have the whole model coloured, we need to assign the texture provided by the set of external images to the rest of the vertices. As was mentioned before, the images are taken without restrictions about the point of view and they may or may not overlap.

**Figure 7.**
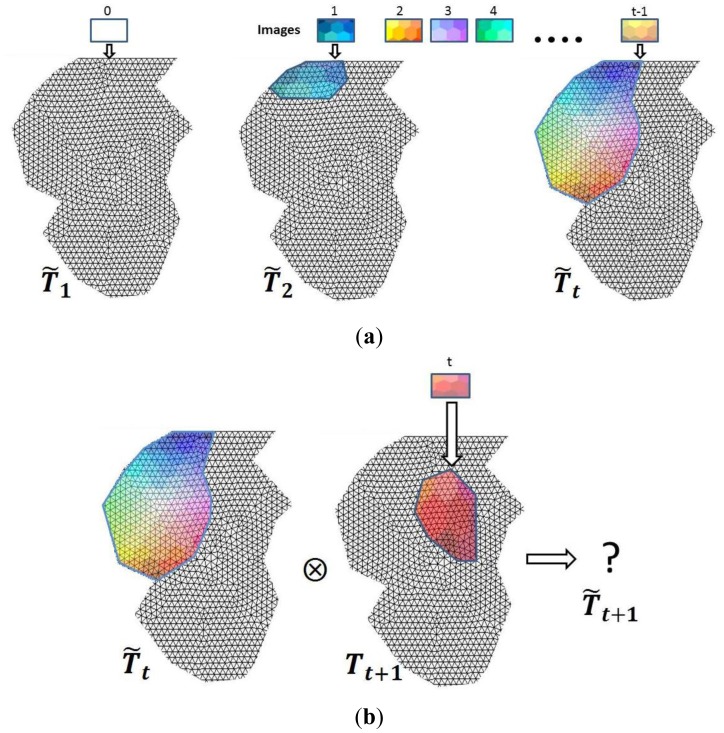
(**a**) Assigning images to the mesh model and evolution of matrix ***T̃****_i_*; (**b**) Integration of a new image.

We carry out an iterative procedure that assigns colour to some of the nodes as new images are matched onto the geometric model. Let us assume a particular step ***t*** in the iterative process. At this point, we denote ***T̃****_t_* as the matrix generated throughout the earlier ***t-1*** iterations which stores the colour information extracted from ***t-1*** external images. A new external image can provide texture for some of the vertices that have not yet been assigned, leading to a matrix ***T****_t_***_+1_** that must be combined with ***T̃****_t_* to produce a new matrix ***T̃****_t_***_+1_** (Figure 7). This process is generically denoted by the symbol ⊗ in Equation (13)

(13) $${\tilde{T}}_{t+1}={\tilde{T}}_{t}⊗T_{t+1}$$

In a sense, matrix ***T̃****_t_* is a matrix accumulated after ***t-1*** iterations (see Figure 7). Let us suppose that we have the mesh model without colour in iteration number 0. The colour fusion process is defined depending on the overlapping between the new view and the current status of the model. Thus if ***T̃****_t_* y ***T****_t_***_+1_** have no nodes in common, Equation (13) then becomes ***T̃****_t_***_+1_ = *T̃****_t_* **∪**.***T****_t_***_+1_** Nevertheless, if ***T̃****_t_* y ***T****_t_***_+1_** have common nodes the colour merging then becomes a more complex process. We can distinguish between global and local approaches.

Depending on the kind of scene we are working on, we apply either one of the two processes described below or both of them. We will denote the two processes using symbols ➀ and ➁ in Equations (14) and (15):

(14) $${\tilde{T}}_{t+1}={\tilde{T}}_{t}1T_{t+1}$$

(15) $${\tilde{T}}_{t+1}={\tilde{T}}_{t}2T_{t+1}$$

**Figure 8.**
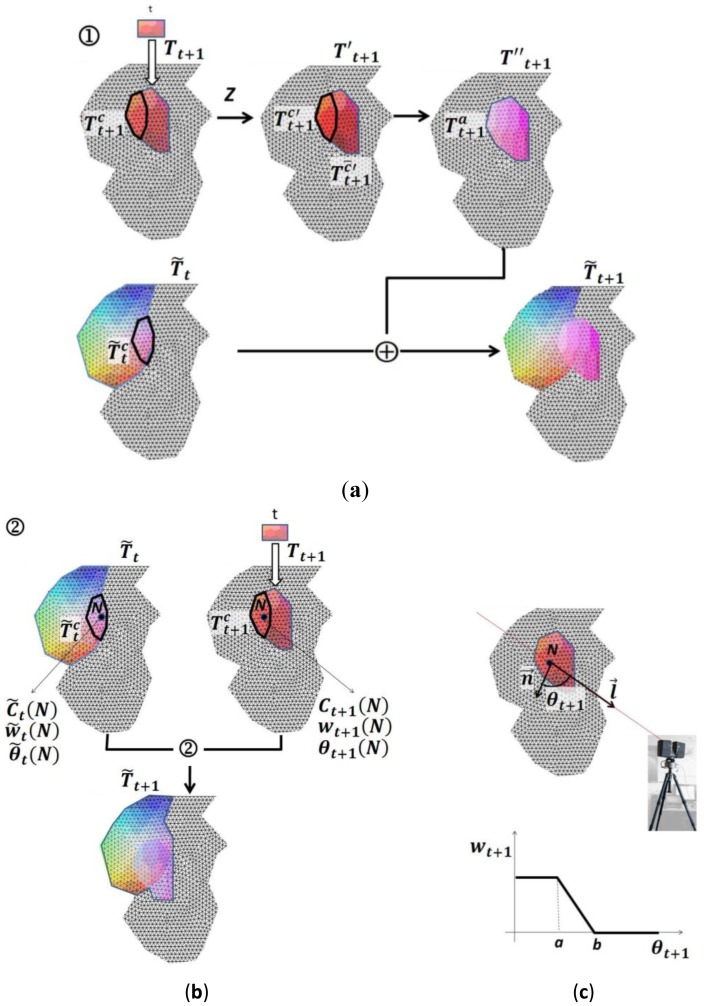
Illustration of multi-view colour-fusion processes ➀ (**a**) and ➁ (**b**).

The chart in Figure 8 illustrates both processes. The process ➀ is illustrated in Figure 8(a). In order to make the notation more readable from here on we will assume that the superscript “***c***” in the following equations will signify the overlapped part of matrices ***T̃****_t_* and ***T****_t_***_+1_** and superscript “***c̄***” will be the non-overlapped part. Thus ${\tilde{T}}_{t}^{c}$ and ${\tilde{T}}_{t+1}^{c}$ are the sub matrixes for the common nodes in matrices ***T̃****_t_* and ***T****_t_***_+1_**.

Process ➀ consists of three steps. First, a transformation is applied over $T_{t+1}^{c}$, and ***T****_t_***_+1_** turns on ***T*′***_t_***_+1_**. This transformation is obtained by using the colour relationship between ${\tilde{T}}_{t}^{c}$ and $T_{t+1}^{c}$, which can be modelled by means of transformation Z in Equation (16):

(16) $${\tilde{T}}_{t}^{c}=ZT_{t+1}^{c}$$

The solution for Z is given by the equation:

(17) $$Z={\tilde{\text{T}}}_{t}^{c}{\left[T_{t+1}^{c}\right]}^{T}{\left[T_{t+1}^{c}\cdot {\left[T_{t+1}^{c}\right]}^{T}\right]}^{-1}$$

Thus, matrix ***T****_t_***_+1_** is transformed after:

(18) $$T_{t+1}^{\prime }=Z\cdot T_{t+1}$$

After this, we calculate the average values between ${\tilde{T}}_{t}^{c}$ and $T_{t+1}^{c\prime }$ and add the non-common part $T_{t+1}^{\bar{c}\prime }$, generating the partial matrix $T_{t+1}^{a}$ (Equation (19)). ***T*′***_t_***_+1_** then turns on ***T***″*_t_***_+1_**.

(19) $$T_{t+1}^{a}=\left\{\left[\frac{{\tilde{T}}_{t}^{c}+T_{t+1}^{c\prime }}{2}\right]\cup T_{t+1}^{\bar{c}\prime }\right\}$$

Finally, $T_{t+1}^{a}$ is inserted in ***T̃****_t_* and ***T̃****_t_***_+1_** is generated. We denote the insertion operator by means of the symbol ⊕ in Equation (20):

(20) $${\tilde{T}}_{t+1}={\tilde{T}}_{t}⊕T_{t+1}^{a}$$

Process ➁ is illustrated in Figure 8(b,c). It consists of making a colour substitution of ${\tilde{T}}_{t}^{c}$ by using a weighted expression of the colour. The weight factors are based on the observation angles for the nodes of which the colour values are about to be modified.

We start by defining a weighted observation angle for each node of the model which is seen in previous scans. This parameter will be used if we want to take into account local properties in the model. Formally, we define the weighted observation angle as follows.

Let us suppose ***N*** be a node of ***T̃****_t_* and ***T****_t_***_+1_** with assigned colours ***C̃****_t_*(***N***) and ***C****_t_***_+1_(***N***), r**espectively. Let ***θ****_t_***_+1_**(***N***) be the observation angles of ***N*** in the step ***t***, where $\theta_{t+1}(N)=(\hat{\vec{n},\vec{l}}),\vec{l}={\vec{NR}}_{t+1},\vec{n}$ being the normal at ***N*** and ***R****_t_***_+1_** being the camera position in iteration ***t***. Finally, let ***θ̃****_t_*(***N***) be a weighted observation angle which is computed after ***t-1*** iterations. The weighted observation angle at iteration ***t*** is calculated as follows:

(21) $${\tilde{\theta }}_{\text{t}+1}(\text{N})=\frac{\theta_{\text{t}+1}(\text{N})\cdot {\text{w}}_{t+1}+{\tilde{\theta }}_{\text{t}}(\text{N})\cdot {\tilde{w}}_{\text{t}}(\text{N})}{w_{t+1}(N)+{\tilde{w}}_{t}(N)}$$

in which weights ***w****_t_***_+1_** are based on angles ***θ****_t_***_+1_**:

(22) $$w_{\text{t}+1}(\text{N})=\{\begin{array}{c} 1,\theta_{t+1}(N)\leq a\\ \frac{\theta_{t+1}-\text{b}}{a-b},a<\theta_{t+1}(N)<b\\ 0,\theta_{t+1}\geq b \end{array}$$

and ***w̃****_t_* is based on previous weights in the following manner:

(23) $${\tilde{w}}_{t}(N)=\sum_{k=1}^{t-1}\frac{w_{k}}{t}$$

In Equation (22)***a*** and ***b*** are two thresholds imposed to the observation angles.

With this algorithm, matrix ***T̃****_t_* is modified taking into account the colour and the observation angle for each common node in ***T̃****_t_* and ***T****_t_***_+1_**. The colour updating for a node *N* in ***T̃****_t_* is given by the expression:

(24) $${\tilde{C}}_{\text{t}+1}(\text{N})=\frac{C_{\text{t}+1}(\text{N})\cdot {\text{w}}_{\text{t}+1}(\text{N})+{\tilde{C}}_{t}(\text{N})\cdot {\tilde{w}}_{t}(N)}{w_{t+1}(N)+{\tilde{w}}_{t}(N)}$$

Where ***C̃****_t_*(***N***) is the colour of ***N*** in ***T̃****_t_* and ***C****_t_***_+1_** is the colour of ***N*** in ***T****_t_***_+1_**, the weights ***w****_t_***_+1_** and ***w̃****_t_* are calculated with Equations (22) and (23) from the observation angle *s* ***θ****_t_***_+1_**(***N***) and ***θ̃****_t_*(***N***), respectively.

In conclusion, the process of colour merging, depending on the type of scene we are dealing with, can consist of:

- A global correction plus average following process ➀; that is, applying Equations (16) to (20).
- A local correction depending on the observation angle following process ➁; that is, applying Equations (21) to (24).
- A global correction followed by a local correction, which results in:
  (25) $${\tilde{T}}_{t+1}=({\tilde{T}}_{t}➀T_{t+1})➁T_{t+1}$$

## 5. Experimental Results

**Figure 9.**
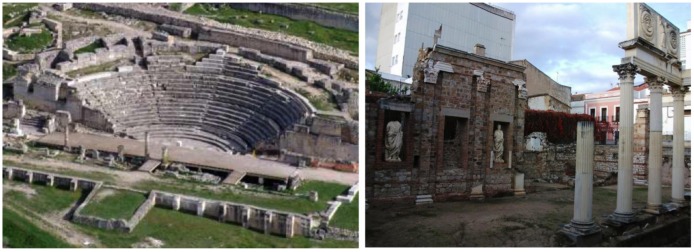
Pictures of the Roman Theatre of Segobriga (**left**) and the *Fori Porticus* of Emerita Augusta (**right**).

In order to illustrate the worthiness of our proposal, we present in this section the results for the reconstruction of two Roman archaeological sites from to the first century A.D., the Roman Theatre of Segobriga and the *Fori Porticus* of Emerita Augusta (Figure 9). They are both good prototypes of exterior scenarios, huge outdoor spaces with a high degree of difficulty due to the multitude of occlusions that arise when acquiring the surfaces.

For the acquisition of the geometry, we used both a Faro LS 880 laser scanner and a Faro Photon 80 sensor to provide a panoramic view of the scene. Azimuth and elevation angles range over [0, 360°] and [−65°, 90°], respectively, and the maximum data density of the scanner is 40.110 × 17.223 points. Obviously when the data is used in outdoor scenes most of the potential data are missed because they correspond to the sky and only a part of the data are useful. The colour information has been obtained with two Nikon D200 cameras with Nikon AF DX Fisheye lens, one for each scanner. This system composes a single panoramic image from 10 photos, 10.2 Mpixels each. The system takes 1 hour and 49 minutes to scan a panoramic sample plus 8 minutes to take the sequence of photos.

### 5.1. The Roman Theatre of Segobriga

**Figure 10.**
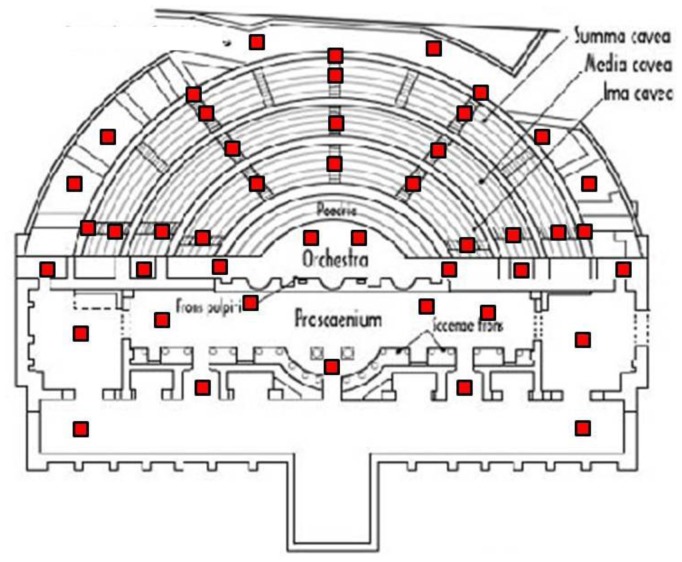
Small red spots point out the scanners positions on a map of the theatre.

As usual, the first task we accomplished *in situ* was the planning of the accessible scanner positions. The volume that could be covered in a single scan is limited by several factors: the field of view, the accessibility of the scanner, the occlusion conditions and the overlap with other scans. The theatre is split into four parts: *Cavea, Poedria, Orchesta* and *Proscaenium*. Most of the scans were taken in the Cavea zone because of the multiple occlusions that the stairs generate everywhere in this area. Figure 10 shows the 56 scanner positions we planned initially on a map of the theatre. But as we took new scans we sometimes detected some redundant views that were discarded. Thus, we ended up taking 45 scans of the theatre.

We worked with resolution of up to 4,010 × 1,723 points per scan. The average amount of data and memory requirement per range image at this resolution were 3,084,840 points. It must be remembered that many data are missed in outdoor scenes. The geometric model building stage was run over an Intel^®^ Core™ 2 6400 2.13 GHz with 2 GB RAM computer whereas in the colour model phase we used an Intel^®^ Pentium™ 4 3.2 GHz, 3 GB RAM.

Apart from noise and outliers filtering tasks in the pre-processing stage, we had to correct errors in colour assignment. The system is capable of associating colour information to each point of the scene but, due to small camera alignment errors, the colour-geometry registration must be refined. To solve this problem we set a pair of corresponding points in reflectance and colour images and aligned accurately.

To begin generating the model, the acquired data have to be registered so that they are all in the same coordinate system. Data registration is a well-studied problem, and methods to automatically register laser scans are commercially available. In order to make the registration process more precise, we carried out an initial alignment of the two scans by manually marking related points in the two datasets to be registered. After this, the ICP algorithm was run. Finally, with the aim of having as regular and homogenous a model as possible, we performed normalization and resampling work. Table 1 shows computational times for different steps in the creation of the geometrical model and for several resolutions of the scanner. Resolution R1 corresponds to 4,010 × 1,723 = 6,909,230 points per scan, and R2 to R7 are resolutions that go from 90%R1 to 40%R1. The filtering processes eliminate erroneous points provided by the scanner: points generated in the infinite (spread points), noise (owing to reflections and shiny surfaces) and outliers (points outside the scene). Registration signifies the process of putting in correspondence two overlapped surfaces. After this process a coarse transformation is achieved. The registration refinement stage calculates an accurate alignment between both surfaces. Finally, the merging stage samples the points on the surface and rewraps it in order to make smooth transitions and generate only one cloud of points.

**Figure 11.**
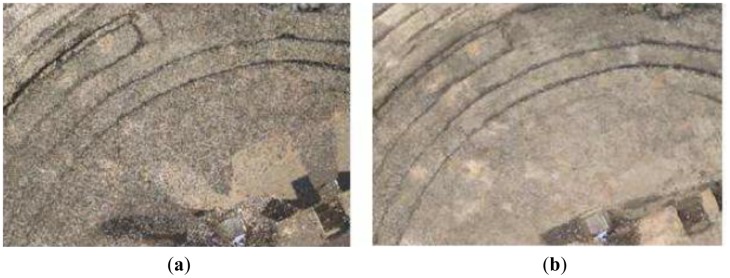
Erroneous colour fusion made with commercial software (**a**) and the result after applying our algorithm (**b**).

**Figure 12.**
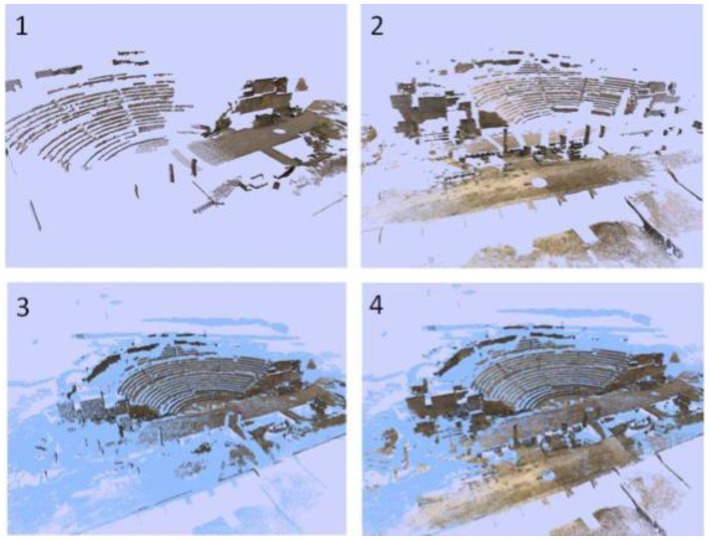
(**1**–**2)**. Two coloured scans, (**3)** Matching of coloured scan 1 onto the geometrical model, (**4)** Result after merging scan 2 with the current coloured model.

As we have said, one of the most common failures of current conventional 3D sensor devices is that they are unable to make a realistic colour fusion between overlapped samples. To overcome this drawback, we used our colour fusion algorithm in the geometric model generated in the previous phase. Figure 11(a) displays an example of a deficient colour assignment carried out with on-line images which were processed using commercial software. Commercial modelling tools are currently inefficient and do not provide an acceptable texture from different light conditions and scanner positions. This partially can occur owing to the lack of information that the software manages when the colour is assigned to the model. For example, the position of the scanner with respect to the surface of the object might be unknown if the 3D data are loaded from a global reference system. In the majority of the cases, the colour of overlapped nodes from different viewpoints is assigned drastically (RapidForm, Faro Scene). Thus, sometimes the colour of overlapped zone in the last scan replaces the existing colour in the model or, at the much, a simple average is calculated. Apparently, there are no filtering and colour inconsistency detection processes so that the final colour is erroneous. Figure 11(b) shows the result using external off-line images and the colour fusion process explained here. Our solution, as explained throughout the paper, follows a sequential merging strategy that entails three stages: global processing, local processing and filtering. Figure 12 illustrates how the geometric plus colour model is sequentially updated as the colour information of a new scan is integrated.

**Figure 13.**
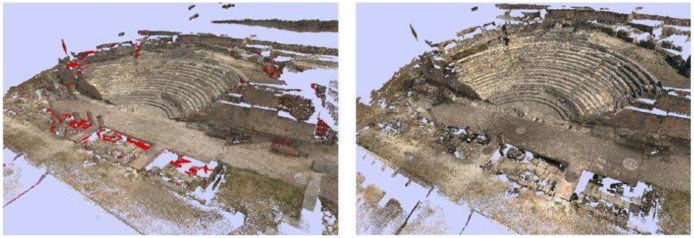
Colour holes depicted in red (left); Result for the nearest neighbour filling algorithm run on them (**right**).

Colour discontinuities corresponding to typical seam or edge points arise after local correction. The solution to such types of colour discontinuities is addressed here by means of a linear smoothing filter in a 3D environment. We applied a 3D discrete Gaussian filter over the three colour components. After merging all scans several colour-holes may appear. This is due to the fact that the colour merging algorithm imposes several restrictions that a few points do not satisfy. We solved the colour-hole problems following a simple nearest neighbour interpolation algorithm. Figure 13 shows the colour-holes detected in the model in red and the result after the filling process. Finally, we generated the colour mesh model in which the colour of the patch is assigned by means of a bicubic interpolation over the vertices of the patch. Table 2 presents computational cost, memory requirement and number of filled holes concerning the colour assignation stage. The table includes several mesh model resolutions. The primary complete mesh-model was a 746 MB file with 9,587,505 triangular patches and average edge of 1.6 cm. *N_p_* symbolizes the number of nodes of the mesh model (in million units). T_1_, T_2_ and T_3_ concern loading, colour merging and colour-hole filling computation times, respectively. The next column (RAM) is the amount of memory required and *N_h_* corresponds to the number of filled patches.

### 5.2. The Fori Porticus of Emerita Augusta

**Figure 14.**
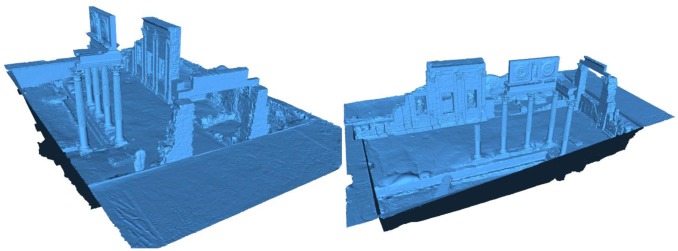
Two views of the final geometrical model for the *Fori Porticus* of Emerita Augusta.

Following the same procedure as for the theatre model, we also generated a complete geometrical model for the *Fori Porticus* of Emerita Augusta in Merida that can be seen in Figure 14. We worked with resolution up to 5,014 × 2,153 points per scan. This site needed 25 scans to be completely reconstructed, each having an average of six million points. These data were taken during four sessions, eight hours per session. The final point cloud of the definitive model was reduced to 48,560,845 points, with a memory size of 1.148 GB. To integrate the texture in the model we took, on a different day and over one hour, 126 shots of the site and applied our method. We chose a cloudy but light day so that illumination conditions were the best. The model was run over on two 3.2 GHz, 4 GB RAM personal computers and a graphic server with two processors, six cores each, two threads per core and 32 GB RAM.

Tables 3 and 4 present approximate computational times for different stages and scanner resolutions concerning the geometrical model generation and colour assignation processes respectively. Resolution R1 corresponds to 5,014 × 2,153 points per scan, and R2 to R7 are resolutions that go from 90%R1 to 40%R1. Table 3 shows, besides the times concerning the registration between two range images, the time required to obtain the whole mesh model (“Meshing”) and the time required to normalize (edge regularization) and refinement (mesh smoothing). Table 4 provides details of specific steps referred to in Sections 2, 3 and 4: extraction of the ortho-image from the 3D model, correspondence between the model and the external image, colour filling process and colour merging in the whole coloured model.

**Figure 15.**
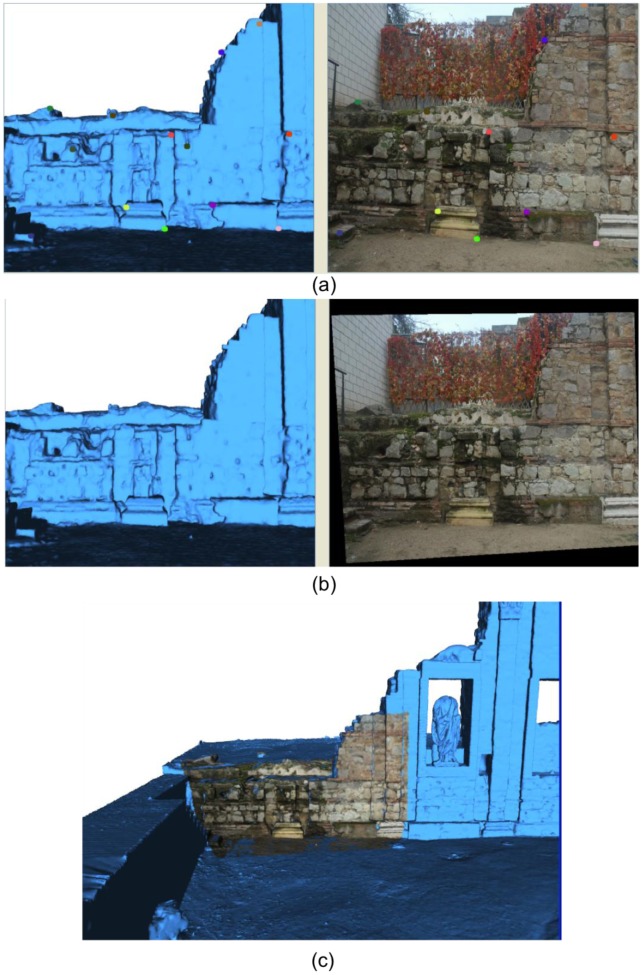
(**a**) Selection of the corresponding pairs on the 3D geometrical model ortho-image and the external image; (**b**) Result of the colour transformation method on the ortho-image (left) to obtain the coloured ortho-image (right); (**c**) Colour information mapped onto the 3D model.

**Figure 16.**
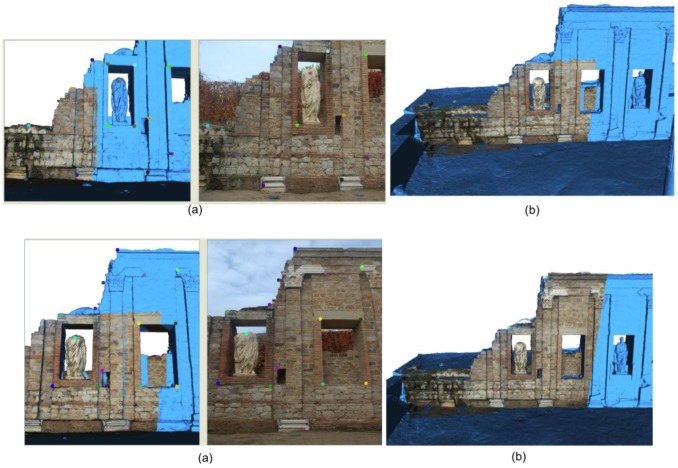
(**a**) Selection of corresponding pairs; (**b**) Colour information mapped onto the 3D model.

**Figure 17.**
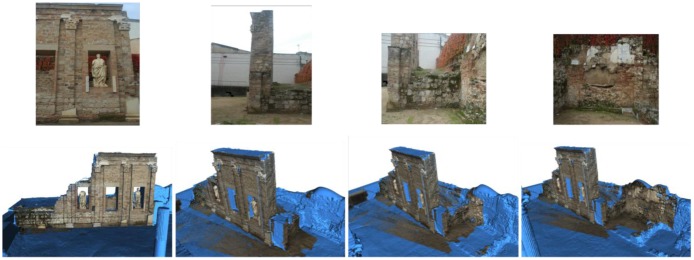
External images taken under appropriate environmental conditions (**top**); Evolution of the coloured model as the colour information is added (**bottom**).

**Figure 18.**
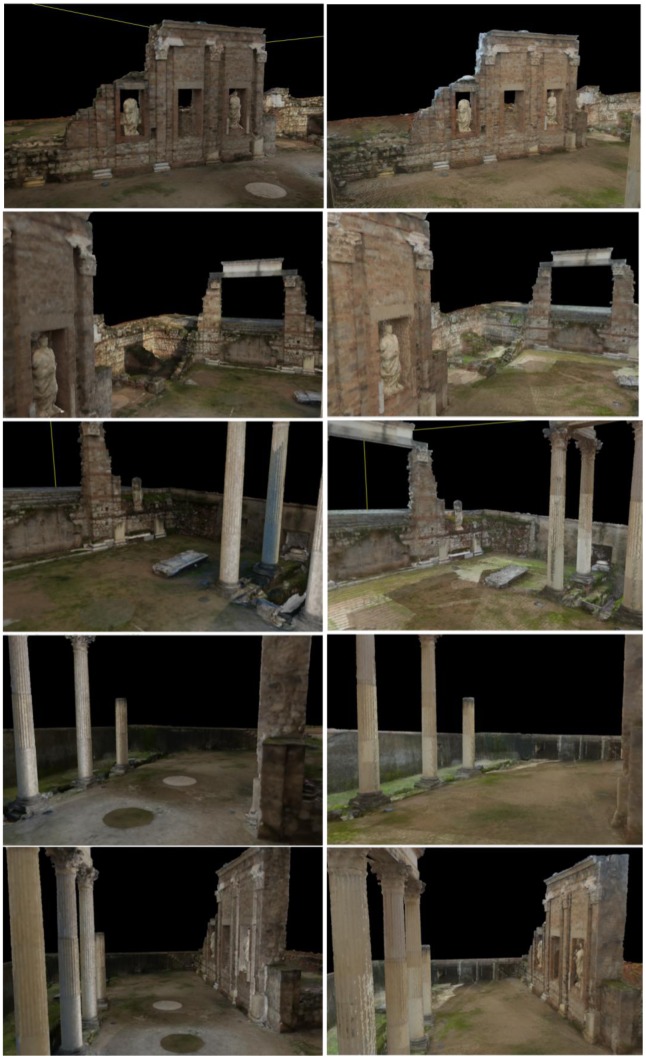
Pictures of a previous coloured model generated from the images provided for the scanner on-board camera (**left**) in comparison with pictures of the photorealistic model created from the geometrical model and a set of external images taken without any restrictions (**right**).

Figures 15 to 18 highlight some moments of the sequence of steps carried out to assign colour from external images to the geometrical model built.

Finally, we present a set of pictures to show the results obtained after applying our method to the *Fori Porticus* of Emerita Augusta in comparison to the previous coloured model generated by using the colour information provided by the scanner on-board camera (Figure 18). The improvement in the colour model generated from external images can clearly be seen.

## 6. Conclusions

Building accurate and complete (geometry+texture) 3D models of large heritage indoor/outdoor scenes from scanners is still a challenging and unresolved issue on which many researchers are currently working [27,28]. In the last decade technology relating to 3D sensors has significantly improved in many respects and the devices are now more flexible, more precise and less costly than in the past. Consequently, we believe that better reverse engineering results will be obtained in this area. Processing of information, nevertheless, may be inefficient if the scene is digitalized under unsuitable conditions. This frequently occurs in outdoor scenarios in which the illumination and the environment conditions of the scene cannot be controlled. This paper proposes to improve the existing modelling techniques by decoupling the colour integration and geometry reconstruction stages. Colour assignation in particular is carried out as an independent and controlled process. The method has basically two parts: colour assignation for one shot of the scanner and colour fusion of multiple views.

Colour assignation focuses firstly on modifying low-quality coloured 3D models by using sets of images of the scene that have been taken at different times and circumstances than those of the geometrical information capture phase. In this approach a colour transformation is obtained after matching the model's orthoimages with external images. The second line is addressed to directly assigning external images to the complete geometric model. Both approaches are quite simple and can easily be implemented in practice.

The merging colour algorithm is proposed in the last part of the paper. This stage is carried out under an iterative process in which the next external image is automatically integrated into the ‘as-built’ model. The result is a complete coloured model that can be refined in the last step.

The proposed method has been widely tested in large outdoor scenes with very good results; the present paper shows the results obtained at two popular heritage sites in Spain. We have achieved high-quality photorealistic models in all tests we have performed. Comparison with former models is also illustrated at the end of the paper.

## Figures and Tables

**Table 1.** Average computational times in different processing phases of theTheatre of Segobriga geometrical model and for several scanner resolutions.

**Process**	**R1**	**R2**	**R3**	**R4**	**R5**	**R6**	**R7**
Spread points filtering (one scan)	21s	20s	20s	16s	16s	13s	12s
Outliers and noise filtering (one scan)	6s	6s	5s	5s	5s	3s	3s
Normalization and resampling (one scan)	49s	40s	34s	22s	12s	9s	8s
Registration (two scans)	4m35s	3m13s	1m40s	1m14s	1m14s	1m14s	1m14s
Registration refinement (two scans)	4m59s	7m11s	6m33s	5m49s	5m04s	4m35s	3m31s
Merging (two scans)	21s	21s	20s	17s	16s	15s	15s
							

**Table 2.** Interesting figures for several Theatre of Segobriga models.

***N_p_***	**T_1_**	**T_2_**	**T_3_**	**RAM**	***N_h_***
2,93	30s	1h	1h 53m	115 MB	32524
6,02	53s	1h 21m	3h 55m	195MB	61096
7,88	1m 10s	1h 47m	5h 20m	143MB	82959
9,58	1m 28s	2h 10m	8h 5m	298MB	102314

**Table 3.** Computational times for different stages and scanner resolutions of the *Fori Porticus* geometrical model.

**Process**	**R1**	**R2**	**R3**	**R4**	**R5**	**R6**	**R7**
Registration (two scans)	2m	1m42s	1m33s	1m24s	1m22s	1m19s	1m20s
Registration refinement (two scans)	1m	56s	50s	44s	42s	37s	33s
Meshing (complete model)	30m	27m	24m	21m	18m	15m	12m
Normalization and refinement of the final mesh (complete model)	8h	7h	5h	3h	1h	30m	10m

**Table 4.** Computational times for several steps and scanner resolutions in the *Fori Porticus* coloured model building.

**Stages**	**R1**	**R2**	**R3**	**R4**	**R5**	**R6**	**R7**
Pre-processing of each view (normal calculation, format conversion, *etc.*)	5 m	4m30s	4m	3m30s	3m	2m30s	2m
Definition of the ortho-image from the 3D model	2m	1m47s	1m35s	1m23s	1m13s	1m2s	45s
Definition of corresponding pairs between the model and the external image	3m	3m10s	3m20s	3m30s	3m45s	4m5s	5m
New colour modification/assignation	1m	56s	50s	43s	35s	31s	23s
Filling of colour-holes	10m	9m6s	8m10s	7m15s	6m25s	5m30s	4m
Merging of colour with the whole coloured model	3m	2m40s	2m20s	2m	1m49s	1m36s	1m10s
